# Premature ejaculation prevalence among young men who have sex with men: a cross-sectional study with internet users in the metropolitan region of São Paulo, Brazil

**DOI:** 10.1093/sexmed/qfac016

**Published:** 2023-03-01

**Authors:** Margareth de Mello Ferreira dos Reis, Eduardo Augusto Corrêa Barros, Leonardo Monteiro, Cristiano Linck Pazeto, Willy Roberto Camargo Baccaglini, Sidney Glina

**Affiliations:** Sexual Medicine Outpatient Clinic, Urology Department, Faculdade de Medicina do ABC, Santo André (SP), Brazil; Sexual Medicine Outpatient Clinic, Urology Department, Faculdade de Medicina do ABC, Santo André (SP), Brazil; Urology Department, Faculdade de Medicina do ABC, Santo André (SP), Brazil; Urology Department, Faculdade de Medicina do ABC, Santo André (SP), Brazil; Urology Department, Faculdade de Medicina do ABC, Santo André (SP), Brazil; Urology Department, Faculdade de Medicina do ABC, Santo André (SP), Brazil

**Keywords:** premature ejaculation, diagnostic self-evaluation, sexual behavior, sexual health, sexual and gender minorities, cross-sectional studies, rapid ejaculation, prevalence of premature ejaculation, men who have sex with men, e-survey, homosexual, gay

## Abstract

**Background:**

There are only a few studies about the prevalence and correlates of premature ejaculation (PE) among men who have sex with men (MSM).

**Aim:**

(1) To estimate PE prevalence according to 3 assessment methods: self-reported time from penetration to ejaculation (ejaculation latency time [ELT]); Premature Ejaculation Diagnostic Tool (PEDT); and a direct question about the self-perception of ejaculation as being normal, too early (premature), or retarded. (2) To assess the agreement of the 3 assessment methods and identify factors associated with PE according to each method and their combination.

**Methods:**

We evaluated data from 226 MSM who participated in a cross-sectional study about sexual behavior among men living in the metropolitan region of São Paulo, Brazil. They responded anonymously to an online survey between May 2019 and March 2020. We calculated the agreement of the 3 assessment methods and their association with other characteristics using logistic regression models.

**Outcomes:**

Outcomes included the prevalence of PE according to the assessment methods and the association measures (PE vs sociodemographic characteristics and sexual behavior).

**Results:**

The prevalence of PE among MSM was 21.2% (95% CI, 16.1%-27.1%) according to the PEDT, 17.3% (95% CI, 12.6%-22.8%) per self-report, and 6.2% (95% CI, 3.4%-10.2%) by estimated ELT ≤2 minutes. The agreement among the 3 assessments was fair (kappa, 0.31; 95% CI, 0.25-0.37; *P* < .001). Association with PE varied by assessment method: obesity and shorter time for ejaculation with anal sex vs masturbation were associated with PE according to the PEDT and ELT but not self-evaluation. Perception about ideal time to ejaculate ≤5 minutes increased the chance of PE based on ELT. Higher chances of self-reported PE were associated with trying to hold back ejaculation, and lower chances were associated with higher frequencies of masturbation.

**Clinical Implications:**

Combining tools to investigate PE allows the identification of characteristics associated with this condition and may result in improvement in the care of MSM.

**Strengths and Limitations:**

This anonymous online survey provided the privacy necessary for participants to respond freely about sensitive questions, with a low risk of social adequacy bias. However, as it was a secondary analysis of a larger study, it could not evaluate comorbidities (eg, erectile dysfunction, prostatitis, depression) and the use of condoms.

**Conclusion:**

The prevalence of PE among MSM is high and varies according to the instrument used for the assessment, and the agreement among the 3 assessments was only fair.

## Introduction

Sexual dysfunctions are frequent problems in male health^1^ causing suffering to men and their partners.^2^^,^^3^ However, not being heterosexual is often an exclusion criterion in epidemiologic studies on sexual health,^4^ and as a result, there are few studies on sexual dysfunctions in homo-, bi-, or pansexual men who have sex with men (MSM), who may represent almost 5% of the population. In a large cross-sectional study in several Brazilian cities in 2000, 3.9% (95% CI, 2.9%-5.1%) of men aged ≥18 years declared themselves as homosexual and 4.7% (95% CI, 3.6%-6.0%) as bisexual.^1^ In Sweden in 2020, 1.9% (95% CI, 1.5%-2.4%) and 2.4% (95% CI, 2.0%-3.0%) of men aged 18 to 84 years were homosexual and bisexual.^2^

Premature ejaculation (PE) is one of the most frequent male sexual dysfunctions, and it may cause impacts on the health of MSM too. The fifth edition of the *Diagnostic and Statistical Manual of Mental Disorders* (*DSM-5*), from the American Psychiatric Association, defines PE as “a persistent or recurrent pattern of ejaculation occurring during partnered sexual activity within approximately 1 minute following vaginal penetration and before the person wishes it”.^3^ The International Society for Sexual Medicine proposed a distinction between lifelong PE (present from the first sexual intercourse in all or almost all relationships, with a latency time to ejaculation ≤1 minute) and acquired PE (reduction in latency time to uncomfortable and clinically significant ejaculation for ≤3 minutes).^4^ In reviewing its guidelines for the management of PE, the society included 2 other PE subtypes, as originally proposed by Waldinger et al:^5^^,^^6^ the “natural variable premature ejaculation,” which was defined not as a typical syndrome but rather a cluster of inconsistent symptoms of rapid ejaculation that occurs without a timeline pattern and belongs to the normal variability of sexual performance, and the “premature-like ejaculatory dysfunction,” in men who experience PE while having normal or even long ejaculation latency time durations.^7^

However, these definitions clearly do not include sexual practices other than vaginal penetration or sexual relationships between MSM. Due to this heterosexist bias of the current PE definitions,^8^ the questionnaires validated for the investigation of patient-reported outcomes of PE assess the symptoms of PE and the psychological and relational consequences only in situations of vaginal penetration; therefore, they are not automatically applicable for MSM.^9^

Ejaculation latency time (ELT) is a way of investigating PE, and it is largely used in studies that include only vaginal intercourse.^10^ It can be measured with a chronometer or estimated by the man. However, this measurement in the literature has also been taken among men who have sex with women, during vaginal intercourse. Just in the last decade, researchers started to propose that different measurement methods of ELT should be used for oral sex, vaginal intercourse, and others. They argue that these sexual activities are different technically, psychologically, culturally, and religiously.^11^ Studies have additionally started to assess ELT in vaginal or anal penetration to include heterosexual couples who practice anal sex and homosexual couples.^12^ In its website, the National Health System in the United Kingdom considers that “it’s up to each couple to decide if they’re happy with the time taken—there’s no definition of how long sex should last.”^13^

Few studies have investigated the prevalence of PE among MSM, who may be considered a hard-to-reach population.^14^ Due to this difficulty, most of these studies are conducted exclusively via the internet,^15–23^ with invitation letters by mail with instructions to fulfil an e-survey^2^ or with online and face-to-face questionnaires in venues frequented by homosexual men.^24^^,^^25^ The heterogenous characteristic of studies—with different populations (homosexual, homo- and bisexual, homosexual with AIDS), nomenclature used to define them (gay, homosexual, bisexual, and MSM), time threshold for PE definition, and instruments used for assessing the condition (Table 1)—produces highly variable estimations of the prevalence of PE among MSM (4.5%-34%) and the characteristics associated with this condition in MSM.^15^^,^^16^^,^^20^^,^^21^^,^^23^ The hypotheses in this study are as follows: (1) the PE prevalence among MSM varies according to the instrument used for the evaluation, and (2) because the different instruments for PE evaluation emphasize different aspects of the syndrome (time to ejaculate, control over ejaculations, suffering or stress), the personal characteristics associated with PE vary by the instrument used by researchers.

Given the scarcity of studies on the prevalence of PE among MSM, as well as the varied estimates of the prevalence and multiplicity of factors associated with PE, the objectives of the present study were (1) to estimate the prevalence of PE among MSM who are internet users according to 3 methods of investigation, (2) to assess the agreement among these methods, and (3) to investigate the factors associated with this sexual dysfunction as assessed by each method and their combination.

The 3 methods of investigation are as follows: ELT according to the participant’s memory; a standardized questionnaire developed to investigate PE; and a direct question about the self-perception of ejaculation as being normal, too early (premature), or retarded.

The research questions (RQ) are as follows:

*RQ1:* Does the prevalence of PE vary in the MSM population according to the assessment method used?

*RQ2:* Is the agreement among the 3 investigation methods low, as it happens in studies with heterosexual populations?^26^

*RQ3:* Do the characteristics associated with PE vary according to the method used to identify the condition?

**Table 1:** Cross-sectional studies about premature ejaculation among men who have sex with men, 2005–2020.

**Year**	**First author**	**Country**	**Target population**	**Method of data collection**	**Method of recruitment**	**Time of PE occurrence**	**Age, y**	**No.**	**PE assessment**	**Prevalence rate, % (95% CI)**
2005	Bancroft^15^	US	Gay men (vs heterosexual men)	Web-based survey	Invitation on a sexuality institute research site	Whole life	20-79	1196	“In your sexual activities with a sexual partner, have you ever had a problem in ejaculating (ie ‘coming’) too quickly?” Answers: never, occasionally, less than half the time, most of the time.	Most of the time: 4.5 (3.4-5.8)
2008	Lau^24^	Hong Kong	MSM	Web-based survey and face-to-face interviews	Venue based (including saunas, bars, particular beaches) and internet based	12 mo before the study	18-60	411	“Have you experienced premature ejaculation for at least 3 consecutive months in the preceding 12 months when having sex with MSM partners?”	10.4 (7.7-13.8)
2010	Hirshfield^16^	US	MSM	Web-based survey	Eight gay-oriented websites in the US and Canada, ranging from sexual networking and chat to news sites, hosted banners linking to the questionnaire	Past 12 mo	18-85	7001	“Was there ‘a period of time’ in the past 12 months during which you had an orgasm too quickly (premature ejaculation)?”	34.0 (32.0-35.1)
2011	Shindel^17^	English-literate men from the US, Canada, Europe, and Australia	MSM	Web-based survey	Invitation to local, national, and international LGBT community centers; organizations catering to MSM; and advertisements on Facebook		30-79	1361	PEDT ≥9	Range: 11-27 per age and HIV status (95% CIs not calculable)
2012	Shindel^18^	English-literate men from the US, Canada, Europe, and Australia	MSM	Web-based survey	Invitation to local, national, and international LGBT community centers; organizations catering to MSM; and advertisements on Facebook	Not available	18-81	2640	PEDT ≥9	Range: 8-12 among decades of life (95% CIs not calculable)
2013	Vansintejan^19^	Belgium	MSM HIV+	Web-based survey	Distribution of 25 000 flyers during various gay events and hyperlinks to the questionnaire placed on multiple, mostly gay-orientated, websites	4 wk before the study	18-88	72	Index of Premature Ejaculation	18.0 (10.0-28.9)
2014	Tsui^25^	China	MSM	Web-based survey and face-to-face interviews	Venue based (including saunas, bars, particular beaches) and internet based	Past 12 mo	≥18	519	“Have you experienced premature ejaculation for at least 3 consecutive months in the preceding 12 months when having sex with MSM partners?”	19.3 (16.0-22.9)
2015	Ivankovć^20^	Croatia	Heterosexual and nonheterosexual men	Web-based survey	Heterosexual: banner notices posted on general health information, men’s health, and online dating websites; nonheterosexual: LGBT organizations and Facebook	Preceding 12 mo	18-50	Heterosexual: 933. Nonheterosexual: 561	National Survey of Sexual Attitudes and Lifestyles	Heterosexual: 26.8 (24.0-29.8). Nonheterosexual: 18.9 (15.7-22.4)
2015	Peixoto^21^	Portugal	Gay men	Web-based survey	The online survey was publicized on several Portuguese LGBT forums, websites, and social networks (focused recruitment)	6 mo before the study	18-68	425	PE scored latency time “less than 60 seconds” (self-reported)	12.5 (9.4-15.6)
2019	Levitan^22^	US and Canada	Gay and bisexual men	Web-based survey	Several Reddit forums dedicated to men, women, LGBT, and fitness- and weight loss–related topic issues	Not available	18-40	185	PEDT ≥11	11.9 (7.6-17.4)
2019	Grabski^23^	Poland	Homosexual and bisexual men	Web-based survey	Announcements placed on health- and lifestyle-related websites, including those devoted to men’s sexual health and directed at the nonheterosexual audience	Not available	18-70	Homosexual: 1044. Bisexual: 442. Total: 1486	PEDT ≥11	Homosexual: 11.8 (10.0-14.0). Bisexual: 14.5 (11.3-18.1). Total: 12.6 (10.4-14.9)
2020	Björkenstam^2^	Sweden	Heterosexual, bisexual, and homosexual men	Paper questionnaires were mailed with an information letter on the survey and its purpose	Simple stratified random sample of Swedish national men	Past 12 mo	16-84	Heterosexual: 5832. Bisexual: 119. Homosexual: 94	“Have you experienced as a problem have had an orgasm quicker than I wanted to?”	Heterosexual: 17. Bisexual: 17. Homosexual: 12. (Weighted prevalences without 95% CI)

Abbreviations: LGBT, lesbian, gay, bisexual, and transgender; MSM, men who have sex with men; PE, premature ejaculation; PEDT, Premature Ejaculation Diagnostic Tool.

**Table 2:** Sociodemographic characteristics of men who have sex with men responding to the survey. São Paulo metropolitan region, Brazil, 2020 (*n* = 226).

	**No.**	**%**
Age, y		
≤19	59	26.1
20-29	134	59.3
≥30	33	14.6
Race/ethnicity		
White	146	64.6
Other	80	35.4
Education		
Up to high school	48	21.3
Undergraduate: incomplete	102	45.1
Undergraduate: bachelor	50	22.1
Postgraduation: graduate	26	11.5
Activity		
Working	128	56.6
Studying	89	39.4
Unemployed and not studying	9	4.0
Income,^a^ US $		
No income: dependent on others	62	27.6
≤1175	128	56.9
1176-2355	18	8.0
≥2356	17	7.5
No information	1	
Sexual orientation		
Homosexual	144	63.7
Bisexual	71	31.4
Pansexual	11	4.9
Stable relationship		
No	110	48.7
Yes	116	51.3
Obesity		
No	189	83.6
Yes	37	16.4
Physical activity		
No	101	44.7
≤2/wk	54	23.9
≥3/wk	71	31.4

^a^Equivalent to 5 times the Brazilian minimum wage in 2019, corresponding to US $ 1175.00, exchange date: February 3 2020.

## Methods

### Study design, ethics, setting, and reporting

This cross-sectional study was approved by the Institutional Review Board of our university hospital. Participants of this online survey were men aged ≥18 years living in the metropolitan region of São Paulo, Brazil. They signed informed consents electronically before responding. In this consent form, we explained the study objectives and that there were no right or wrong answers for the questionnaire, which was designed to capture their perceptions and experiences only. No personal information was asked, and participants responded anonymously. We report this study following the STROBE (Strengthening the Reporting of Observational Studies in Epidemiology) and CHERRIES (Checklist for Reporting Results of Internet E-surveys) guidelines.

### Participants and study size

This article reports on a subsection of a cross-sectional study conducted online about PE, for which we collected data from May 22, 2019, to March 3, 2020.^26^ Briefly, we conducted an anonymous free-access e-survey with a nonprobabilistic convenience sample.^27^ We included men reached through our university and the researchers’ social media channels (especially Facebook and WhatsApp). We did not use monetary or other incentives to stimulate survey participation and did not disseminate the research on sites or social media channels specialized in gay or homosexual audience. In 2019, about 77% of the population in the metropolitan region of São Paulo had access to the internet.^28^ In the study reported here, we analyze a subsample of adult men who declared having sex with men (MSM): homosexual, bisexual, or pansexual. We did not include heterosexual men in the current analysis.

Because this analysis is made on a subsample of participants of another study,^26^ we had to recalculate the sample size to minimize the probability of type I and II errors. A minimum sample size was calculated to estimate the prevalence of PE among MSM based on the following parameters: prevalence of PE, 18% (estimated from the results of studies that included MSM recruited from the internet)^21^^,^^25^; error margin (accuracy), 5%; and confidence limit (α), 5%. A minimum sample size of 227 was reached. The size was calculated with OpenEpi version 3.01 software (Open Source Epidemiologic Statistics for Public Health).

A total of 829 men responded to the questionnaire online during the study period (most during February 2020, after we intensified recruitment posting through social media). Initially, we excluded 264 men who lived out of the São Paulo metropolitan region, 337 heterosexual men, 1 man who was not yet 18 years of age, and 1 man who had answered the questions about sexual behavior in an inconsistent way. All 226 remaining questionnaires had complete responses, so we did not need to use imputation for any variable.

### Questionnaire

For data collection, we used the Google Forms platform, with an initial section with questions about date of birth and area of residence. These allowed us to exclude participants who lived outside the metropolitan region of São Paulo (that includes several cities in a commuter belt anchored by the capital) or were not adults. All items in the survey were made “required,” and the final results were recorded only if the participant answered all questions.

Some participants accessed the university’s or researchers’ Facebook pages, and others received the link for the questionnaire on their WhatsApp (and could send the link to another person). The link gave access to the questionnaire in the Google platform.

We did not randomize the questionnaire items, as the questionnaire was short (33 questions), and we did not use adaptive questioning. The mean number of items (questions) per page was 4.7, and the questionnaire had 7 screens. All questions were mandatory. Participants could not check the consistency of answers before submitting the questionnaire. They could review their answers through a “back” button.

We could not calculate the response rate, as we do not have a denominator. As the participant could submit the questionnaire only if he had answered all questions from the first page to the last, it was also impossible to calculate the completion rate.

We did not use cookies to assign a unique user identifier to each client computer, and we did not use the IP address to identify potential duplicate entries from the same user (we did not store the IP addresses). We could not use, with the resources that we had, other techniques to analyze the log file for identification of multiple entries or the time taken for a response.

We collected information on sociodemographic characteristics such as occupation (or student or unemployed), income (in US dollars; no income, ≤$1175, $1176-$2355, ≥$2356), and race/ethnicity (White or non-White). Age was categorized, in this analysis, as ≤19, 20 to 29, and ≥30 years. Education was classified as up to high school, undergraduate (incomplete), undergraduate (bachelor), and postgraduation (graduate). We also asked the frequency of physical activity (none, ≤2 times per week, ≥3 times per week) and the body height and weight to calculate body mass index and classify the sample according to obesity (≥30 kg/m^2^).

The form had questions about the men’s personal lives: whether they were in a stable relationship (yes or no) and their sexual orientation as homosexual, bisexual, or pansexual. The participants also answered questions related to their sexual habits: frequency of masturbation, situations in which they judged the time for ejaculation to be shorter (during masturbation, oral sex, vaginal sex, or anal sex), existence and time of foreplay before penetration, and time considered ideal for ejaculation by the participant and his partner.

### PE assessment

We investigated PE using a set of 3 tools.

The first was a direct question about the self-perception of ejaculation as being normal, too early (premature), or retarded.

The second assessment was a specific question about ELT as categorized by the participant: ≤1, ≤2, ≤5, ≤15, and >15 minutes. Although the criterion for considering a PE diagnosis per the *DSM-5* is 1 minute, we opted for an ELT ≤2 minutes as a cutoff because reported ELT is usually higher than the time measured with a chronometer.^29^

The final method was the Premature Ejaculation Diagnostic Tool (PEDT),^30^ in its version translated to Portuguese.^31^ The PEDT has 5 questions, with responses scored on a scale of 0 to 4 in each category so that the final score ranges from 0 to 20 points. A score ≤8 is indicative of no PE, 9 or 10 is possible PE, and ≥11 indicates probable PE.^32^ Although the PEDT was designed to investigate PE in heterosexual men, we decided to use it because it is a simple and brief and its questions do not explicitly mention vaginal sex. This instrument has been used by other researchers who investigated PE among MSM.^18^

### Statistical analysis

We exported data from GoogleForms to spreadsheets and excluded participants who declared dates of birth indicating that they were <18 years old or addresses reflecting that they did not live in the metropolitan region of São Paulo or who answered the questions about sexual behavior in an inconsistent way (answer of a question is contradictory with another answer). We also checked the existence of duplicates using the variables of birth date and city of residence. We did not use weighting of items or propensity scores in the statistical analysis. Then we analyzed data using Stata (version 13.1) and considering *P* < .05 as significant.

The main outcome evaluated in this study was the prevalence of PE among MSM according to the 3 investigation methods and their agreement. We calculated absolute and relative frequencies and the 3 prevalence estimates with 95% CIs according to each criterion: ELT ≤2 minutes, PEDT score ≥11, and self-perceived PE. The agreement among them was assessed with the kappa coefficient.^33^

To investigate the association between the sample’s characteristics and the presence of PE according to each investigation method and the combination of methods, we calculated crude and adjusted odds ratios (ORs). As a first step, we evaluated crude ORs (95% CIs) for each characteristic of the sample separately (univariate analysis). For the construction of each final model, we included all variables that had a *P* value ≤.20 in the univariate analysis. We adjusted OR estimates using logistic regression. We used the likelihood ratio test to assess the contribution of each variable to the final model and assessed the model fit using the Hosmer-Lemeshow test.^34^ In the comparison of the different statistical models tested for each outcome, we chose the one with the greatest plausibility from biological and behavioral perspectives.

## Results

### Demographics

The mean age of the 226 MSM analyzed here was 24.7 years (SD, 8.4 ; range, 18-74), and most (85.4%) were young adults, aged ≤29 years (Table 1). The majority were White (64.6%), had completed at least high school (66.4%), and were working (56.6%), although more than half had a low income (56.9%; ≤$1175, exchange date: February 3, 2020) or no income at all (27.6%). Obesity was not highly prevalent, but only 31.4% of participants practiced physical activity frequently.

### Sexual behavior and time for ejaculation

Half of the 226 MSM were in a stable relationship (Table 2). They declared that they masturbated frequently: 45.6% at least 3 times a week and 42.5% every day. The relationship atmosphere was considered good for the majority of men (ie, sexual interplay was considered good), although just 39.4% of them spent a few minutes on foreplay or did not practice any sexual activity before intercourse, as shown in Table 3. Most men said that they tried to hold ejaculation (62.8%) always or sometimes.

**Table 3:** Behaviour and perceptions of the time for ejaculation of men who have sex with men responding to the survey. São Paulo metropolitan region, Brazil, 2020 (*n* = 226).

**General sexual behavior**	**No.**	**%**
Masturbation frequency		
≤1/wk	27	11.9
2/wk to alternate days	103	45.6
Every day	96	42.5
Sexual interplay		
Good	143	63.3
Regular or bad	83	36.7
Time of foreplay before penetration		
Around 1 h	26	11.5
Around half hour	111	49.1
A few minutes or there is no foreplay	89	39.4
Do you try to hold ejaculation?		
No	84	37.2
Yes	124	54.9
Yes always	18	7.9
**Ejaculation time**		
Perception of time until ejaculation during solitary masturbation, min		
>5	77	34.1
3-5	97	42.9
≤2	52	23.0
Situation where the time for ejaculation is less		
Masturbation	128	56.6
Anal sex	56	24.8
Oral or vaginal sex	42	18.6
Perception of ideal time between first penetration and ejaculation, min		
>15	111	49.1
6-15	93	41.2
≤5	22	9.7
Partner’s perception of ideal time between first penetration and ejaculation, min		
>15	111	49.1
6-15	86	38.1
≤5	29	12.8
Perception of time between first penetration and ejaculation, min		
>15	91	40.3
6-15	75	33.2
3-5	46	20.4
2	11	4.9
≤1 or ejaculates before penetration	3	1.3

The time for ejaculation from starting masturbation or from penetration (Table 3) in real life seems to be different from what these men think would be ideal. During masturbation, most respondents (65.9%) said that it takes no longer than 5 minutes for them to ejaculate. In fact, 56.6% said that masturbation is when ejaculation is faster. Although the majority of participants think that the ideal time would be at least 6 minutes for themselves (90.3%) and their partners (87.2%)—and that their partners would agree with this—that time limit actually happens to a lower percentage of men: only 73.5% think that they ejaculate ≥6 minutes after penetration.

### Prevalence of PE

Table 4 presents the prevalence of PE according to the assessment instrument. The table shows that using different methods to estimate the prevalence leads to disparate results. The highest prevalence was found with the PEDT: 21.2% (95% CI, 16.1%-35.1%). If the prevalence of PE is calculated when any of the criteria is present, the prevalence reaches 28.8% (95% CI, 22.9%-35.1%). Yet, only 2.7% (95% CI, 1.0%-5.7%) of men met all 3 criteria simultaneously (ie, for being classified as having PE, the man would need to fulfill all 3 criteria). In addition, 33 men (14.6%) classified their ejaculation as “rapid” but had an ELT >2 minutes (possibly indicating “premature-like ejaculatory dysfunction”).

**Table 4:** Comparison of the prevalence of premature ejaculation among men who have sex with men according to the Premature Ejaculation Diagnostic Tool (PEDT), estimate ejaculation latency time and self-evaluation. São Paulo metropolitan region, Brazil, 2020 (*n* = 226).

	**Prevalence**	
**Criteria**	**%**	**95% CI**
PEDT: premature ejaculation (score ≥11)	21.2	16.1-27.1
Self-evaluated premature ejaculation (ejaculating before wanting to)	17.3	12.6-22.8
Latency time after penetration: ≤2 min	6.2	3.4-10.2
All criteria together	2.7	1.0-5.7
At least 1 criterion	28.8	22.9-35.1

Abbreviation: PEDT, Premature Ejaculation Diagnostic Tool.

### Agreement among evaluation criteria

The agreement between the self-evaluated PE (when participants declared that they ejaculate before wanting to) and the PEDT results was moderate (kappa, 0.48; 95% CI, 0.33-0.62; *P* < .001). However, the agreement between the PEDT and ELT was only fair (kappa, 0.29; 95% CI, 0.14-0.43; *P* < .001), and that among all 3 methods of evaluation was also fair (kappa, 0.31; 95% CI, 0.25-0.37; *P* < .001).

### Association between men characteristics and PE

In the univariate analysis, the characteristics significantly associated with a higher OR of PE based on the PEDT were race/ethnicity not White, obesity, and considering anal sex as leading to a shorter latency time (Table 5). The characteristics associated with a higher chance of PE according to an ELT ≤2 minutes were obesity, perception of ideal time between first penetration and ejaculation ≤5 minutes, and considering anal sex as leading to a shorter latency time (partner’s perception of ideal time ≤5 minutes increases the chance of PE, but the association is not significant).

**Table 5:** Premature ejaculation among men who have sex with men according to each assessment method and association with sociodemographic characteristics, behavior and perceptions about premature ejaculation (univariate analysis). São Paulo metropolitan region, Brazil, 2020 (*n* = 226).^a^

	**1: PEDT ≥11**				**2: ELT ≤2 min**				**3: Self-evaluated PE**				**PE: 1, 2, or 3**			
		**95% CI**				**95% CI**				**95% CI**				**95% CI**		
	**OR**	**IL**	**SL**	***P* value** **^b^**	**OR**	**IL**	**SL**	***P* value** **^b^**	**OR**	**IL**	**SL**	***P* value** **^b^**	**OR**	**IL**	**SL**	***P* value** **^b^**
Age, y				.979				.112				.806				.976
≤19	1				1				1				1			
20-29	1.08	0.51	2.30		5.70	0.72	44.93		0.96	0.42	2.18		0.98	0.50	1.92	
≥30	1.05	0.37	3.01		1.81	1.10	29.97		1.32	0.45	3.87		0.4	0.19	0.84	
Race/ethnicity				.041				.547				.302				.358
White	1				1				1				1			
Other	1.95	1.02	3.73		1.40	0.47	4.18		0.67	0.31	1.43		1.32	0.73	2.39	
Education				.507				.814				.831				.671
≤High school	1				1				1				1			
Undergraduate: incomplete	0.56	0.25	1.23		1.69	0.33	8.48		0.81	0.34	1.92		0.69	0.33	1.44	
Undergraduate: bachelor	0.61	0.24	1.54		2.00	0.35	11.46		0.62	0.21	1.78		0.71	0.30	1.67	
Postgraduation: graduate	0.58	0.18	1.84		0.92	0.79	10.65		0.69	0.19	2.47		0.55	0.18	1.62	
Activity				.656				.677				.673				.945
Working	1				1				1				1			
Studying	0.95	0.49	1.85		0.79	0.25	2.43		0.74	0.35	1.55		0.96	0.53	1.75	
Unemployed and not studying	1.87	0.44	7.96		—	—	—		1.24	0.24	6.34		1.23	0.29	5.18	
Income, US $				.898				.731				.561				.696
No income: dependent on others	1				1				1				1			
≤1175	1.01	0.48	2.12		1.1	0.32	3.71		1.26	0.56	2.82		1.16	0.59	2.27	
1176-2355	1.45	0.44	4.81		0.85	0.09	8.14		1.04	0.25	4.27		1.32	0.43	4.09	
≥2356	0.81	0.20	3.24		—	—	—		0.32	0.04	2.74		0.57	0.14	2.22	
Obesity				.002				.006				.213				.033
No	1				1				1				1			
Yes	3.22	1.24	6.86		4.38	1.42	13.49		1.70	0.73	3.97		2.18	1.05	4.50	
Physical activity				.323				.568				.379				.279
No	1				1				1				1			
≤2/wk	0.66	0.29	1.49		0.45	0.09	2.18		0.76	0.32	1.80		0.76	0.37	1.56	
≥3/wk	0.58	0.27	1.26		0.69	0.20	2.40		0.55	0.24	1.29		0.57	0.29	1.15	
Sexual orientation				.843				.424				.789				.432
Homosexual	1				1				1				1			
Bisexual	1.06	0.55	2.07		0.53	0.14	1.98		1.29	0.62	2.69		1.37	0.74	2.54	
Pansexual	—	—	—		—	—	—		1.17	0.24	5.77		0.60	0.12	2.89	
Stable relationship				.594				.918				.161				.851
No	1				1				1				1			
Yes	0.84	0.44	1.59		0.94	0.32	2.79		1.65	0.82	3.35		1.06	0.59	1.88	
Sexual interplay				.424				.935				.805				.516
Good	1				1				1				1			
Regular or bad	1.3	0.16	2.50		0.95	0.31	2.95		1.09	0.54	2.23		1.22	0.67	2.20	
Masturbation frequency				.261				.079				.015				.018
≤1/wk	1				1				1				1			
2/wk to alternate days	0.48	0.19	1.23		0.42	0.11	1.55		0.29	0.11	0.75		0.31	0.13	0.75	
Every day	0.49	0.19	1.27		0.19	0.04	0.89		0.29	0.11	0.76		0.33	0.14	0.79	
Do you try to hold ejaculation?				.091				.514				.050				.150
No	1				1				1				1			
Yes sometimes	2.17	1.04	4.51		0.90	0.30	2.6		2.77	1.19	6.43		1.88	0.99	3.56	
Yes always	1.20	0.30	4.78		—	—	—		1.90	0.45	8.00		1.41	0.44	4.48	
Time of foreplay before penetration				.125				.863				.232				.155
Around 1 h	1				1				1				1			
Around half an hour	1.14	0.35	3.67		1.68	0.20	14.3		1.29	0.35	4.81		1.07	0.39	2.94	
A few minutes or there is no foreplay	2.15	0.67	6.86		1.81	0.21	15.72		2.22	0.60	8.17		1.87	0.68	5.14	
Perception of ideal time between first penetration and ejaculation, min				.538				.018				.663				.677
>15	1				1				1				1			
6-15	0.70	0.35	1.38		2.93	0.74	11.67		1.23	0.59	2.58		0.91	0.49	1.68	
≤5	0.69	0.21	2.22		8.00	1.65	38.76		1.63	0.53	5.00		1.41	0.54	3.69	
Partner’s perception of ideal time between first penetration and ejaculation, min				.449				.056				.714				.789
>15	1				1				1				1			
6-15	1.47	0.75	2.90		3.19	0.80	12.72		1.36	0.65	2.86		1.24	0.67	2.29	
≤5	0.89	0.30	2.61		5.76	1.21	27.38		1.15	0.39	3.44		1.03	0.41	2.57	
Situation where the time for ejaculation is less				.004				.002				.033				.023
Masturbation	1				1				1				1			
Anal sex	3.39	1.62	7.11		5.94	1.74	20.2		2.76	1.24	6.13		2.49	1.26	4.90	
Oral or vaginal sex	1.91	0.80	4.55		0.76	0.08	6.96		2.05	0.82	5.12		1.79	0.83	3.84	

Abbreviations: ELT, ejaculation latency time; IL, inferior limit; OR, odds ratio; PE, premature ejaculation; PEDT, Premature Ejaculation Diagnostic Tool; SL, superior limit.

^a^chi-squared test.

^b^Dashes (—) indicate *not applicable.*

The characteristics associated with a higher chance of PE according self-evaluation in univariate analysis were trying to hold ejaculation and considering anal sex as leading to a shorter latency time. Frequent masturbation was associated with a lower chance of PE (Table 5). The characteristics associated with a higher chance of PE according at least 1 criterion (PEDT, ELT, or self-evaluation) were obesity and considering anal sex as leading to a shorter latency time; frequent masturbation was associated with a lower chance.

In the final logistic models, PEDT results were associated with obesity and considering anal sex as leading to a shorter latency time (Table 6). PE according ELT ≤2 minutes was also associated with these 2 characteristics and to the perception of an ideal time between first penetration and ejaculation ≤5 minutes. Self-evaluated PE was associated with trying to hold ejaculation, while frequent masturbation decreases the chance of this outcome. Finally, a higher chance of PE according at least 1 assessment method was associated with considering anal sex as leading to a shorter latency time and with a lower frequency of masturbation.

**Table 6:** Logistic regression models of associations among premature ejaculation among men who have sex with men according to each assessment method and sociodemographic characteristics, behavior and perceptions about premature ejaculation. São Paulo metropolitan region, Brazil, 2020 (*n* = 226).

	**1: PEDT ≥ 11**				**2: ELT ≤2 min**				**3: Self-evaluated PE**				**PE: 1, 2, or 3**			
		**95% CI|**				**95% CI|**				**95% CI|**				**95% CI|**		
	**OR**	**IL**	**SL**	***P* value** **^a^**	**OR**	**IL**	**SL**	***P* value** **^a^**	**OR**	**IL**	**SL**	***P* value** **^a^**	**OR**	**IL**	**SL**	***P* value** **^a^**
Obesity				.005				.059				—				.044
No	1				1								1			
Yes	3.12	1.43	6.82		3.38	0.99	11.49						2.21	1.03	4.71	
Masturbation frequency				—				—				.027				.033
≤1/wk									1				1			
2/wk to alternate days									0.28	0.10	0.74		0.33	0.13	0.81	
Every day									0.28	0.10	0.75		0.31	0.12	0.78	
Do you try to hold ejaculation?				—				—				.037				—
No									1							
Yes sometimes									2.87	1.22	6.78					
Yes always									1.97	0.45	8.53					
Perception of ideal time between first penetration and ejaculation, min				—				.042				—				—
>15					1											
6-15					3.12	0.74	13.2									
≤5					8.27	1.50	45.46									
Situation where the time for ejaculation is less				.008				.007				—				.023
Masturbation	1				1								1			
Anal sex	3.28	1.54	7.00		6.56	1.81	23.82						2.31	1.14	4.67	
Oral or vaginal sex	1.97	0.81	4.78		1.01	0.10	9.73						1.80	0.82	3.94	
Goodness-of-fit test (Hosmer-Lemeshow)				.617				.618				.998				.932

Abbreviations: ELT, ejaculation latency time; IL, inferior limit; OR, odds ratio; PE, premature ejaculation; PEDT, Premature Ejaculation Diagnostic Tool; SL, superior limit.

Dashes (—) indicate *not significant.*

^a^likelihood test.

## Discussion

The prevalence of PE among MSM in the present study was quite different according to the assessment methods used, ranging from 6.2% when defined as an ELT ≤2 minutes to 21.2% when a PEDT score ≥11 was used. Verze et al^35^ investigated PE prevalence in the Italian male population aged between 18 and 80 years (regardless of sexual orientation) and also observed differences when using a PEDT score ≥11 (with a 18.5% PE prevalence) and ELT <1 minute (12.4%). Indeed, the different tools for PE evaluation are one of the sources of the wide variability in the prevalence values observed in studies with heterosexual^36^ and MSM^37^ populations, and there is wide debate in the literature about the strengths and weaknesses of each instrument used.^38^

The time between first vaginal penetration and ejaculation was first used in 1994 as the endpoint of a clinical trial to assess the efficacy of an antidepressant for the treatment of PE.^10^ ELT can be measured with a stopwatch, used by either the man or the partner, which can generate anxiety for the man and/or partner and a loss of spontaneity. ELT can also be estimated by the man. Among heterosexual men, the estimated ELT is, on average, 1 minute higher than the ELT measured with a stopwatch,^39^ and for this reason, several authors suggest that the cutoff point for the diagnosis of PE be at least 2 minutes, as in the present study, or ≤3 minutes^4^—not 1 minute as in the *DSM-5* definition of PE.^3^ Studies show that men with and without PE tend to overestimate ELT as compared with measures timed by their partners using the chronometer.^40^

In addition to the possibility of memory biases, as evidenced by the differences between recalled and measured times, ELT can vary in the male population in general across different countries,^41^ probably due to biological and cultural aspects. Patrick et al observed a great overlap in the ELT between men with and without PE (as diagnosed by physicians using *DSM-IV-TR* criteria): while 95% of men without PE had an ELT ≥1.88 minutes, 49% of men with PE had an ELT above this threshold (median ELT in the group with PE).^42^ These and other authors suggest that the evaluation of PE should be based not only on ELT measurement but also on subjective aspects, such as the perception of control over ejaculation and the suffering caused by ejaculating earlier than desired.^42^^,^^43^ One study conducted in Portugal investigated the prevalence of PE among MSM using ELT as an outcome.^21^ PE prevalence, defined as ejaculation before 60 seconds, was 12.5% (95% CI, 9.4%-15.6%), and 27.8% of MSM had moderate to extreme levels of suffering as a result of PE.

The *ICD-11* (*International Classification of Diseases, Eleventh Revision*),^44^ in contrast to the *DSM-5*, does not mention any time cutoff points for ELT, and it conditions PE diagnosis on the absence or little control over ejaculation and on clinically significant stress. Studies were more frequent that estimated the prevalence of PE in MSM using questions about the man’s control over the ejaculation.^2^^,^^15^^,^^16^ Patrick et al^45^ demonstrated that the control over ejaculation, unlike ELT, has direct and indirect effects on sexual satisfaction and ejaculation-related distress in men with PE and is therefore a fundamental concept for characterization of this dysfunction. The control over ejaculation is addressed in 3 PEDT questions:^30^^,^^31^ “How difficult is it for you to delay ejaculation?” “Do you ejaculate before you want to?” and “Do you ejaculate with very little stimulation?” The prevalence of PE in MSM in studies that used the concept of ejaculation control and PEDT ranged from 4.5%^15^ to 34.0%.^16^ In our study, the prevalence measure based on the PEDT was the highest among the 3 methods used, demonstrating the ability of this instrument to capture the perception of loss of control over ejaculation.

A question explicitly mentioning whether the man had PE was used by Tsui et al,^25^ who recruited participants through the internet and in places frequented by MSM. The results obtained in our study (17.3%) are quite similar to theirs (19.3%). The variation in the estimates of the prevalence of PE in our study and in the literature suggests the need to combine instruments for the assessment of PE, as suggested by Jern et al.^46^

In addition to the variation in the prevalence of PE and the low to moderate agreement among the 3 assessment methods, we observed that some personal or behavioral variables were associated with PE depending on the instrument used. Obesity and considering ELT lower in anal sex were associated with a higher chance of PE according to the PEDT and ELT but not with the self-perception of PE. Yet, the perception of an ideal ELT <5 minutes increased the chance of PE only when ELT was used as a diagnostic criterion.

The sexual behavior variables (frequency of masturbation and attempt to hold ejaculation) showed an association with only the subjective assessments, such as the self-assessed PE (a man saying that he suffers from PE when asked directly about his perception of his own ejaculation). Yet, when the PE assessment was performed with the 3 instruments simultaneously (therefore with objective and subjective assessments), obesity, frequency of masturbation, and shorter time to ejaculate during anal sex were the variables significantly associated with the presence of PE. If just the ELT was used, the self-assessed PE would not show up as being associated with some sexual behaviors, such as trying to hold ejaculation. We found no other studies on the association between trying to withhold ejaculation and PE.

Masturbating at least twice a week significantly decreased the chance of self-rated PE. The association between masturbation frequency and PE was not observed in another study that investigated this.^19^

We did not find studies investigating the association between obesity and PE in MSM. Studies with heterosexual populations showed discrepant results: while some authors observed lower proportions of obesity among men with PE,^47^ Song et al^29^ verified an association between higher body mass index and PE. Several studies^35^^,^^48^^,^^49^ did not report an association between nutritional status and PE but did find an association between metabolic syndrome and PE.^50^

In our study, MSM who considered the ideal ELT to be ≤5 minutes were more likely to have PE detected with the definition of ELT ≤2 minutes. In our literature review, we did not find studies that explored the association between ELT expectation and PE among MSM. As the estimated ELT and the ideal ELT depend a lot on human subjectivity, it is possible that cultural and emotional aspects interfere in the estimates made.

Studies that address the health and sexual dysfunctions (including PE) of MSM are less numerous in the literature than those that investigate these aspects in the lives of heterosexual men, and there is less knowledge about the behavior and sexual health of the MSM population. Some authors emphasize, for example, that during sexual intercourse between MSM, the frequency of manual stimulation of the genitals by the man himself or by the partner is much more frequent than anal penetration^51^; therefore, the direct transposition of diagnostic criteria and expected standard behavior of heterosexual men for MSM may not be adequate to address the anatomic psychological, and motivational specifics of sexual relationships among MSM.^52^ Yet, studies show that heterosexual men and MSM have perspectives of the physical aspects of the sexual relationship that can be quite mechanical, with great appreciation of the size of the penis and the ability to maintain an erection.^52^ Dysfunctional sexual beliefs, which reinforce unrealistic expectations on sexual relations, are more present in men with sexual dysfunctions than among those without, regardless of sexual orientation.^53^

The recruitment of participants and data collection were carried out in this study through the internet, without the possibility of evaluation by a doctor or other health professional, which could be seen as a limitation. However, since the late 1980s, face-to-face application of instruments (and later by mail and telephone) has dropped substantially in prevalence studies, while the costs of conducting these studies have grown exponentially, and for these reasons, the internet became the preference for conducting prevalence studies on the most varied topics.^54^ In line with the advance in the use of the internet as a source of health information, the European Society for Sexual Medicine published a position in 2020 on the use of the internet to carry out research on health. In the statement, the organization argues that hard-to-reach populations, such as transgender adolescents or those whose living sensitive issues, benefit greatly from surveys performed in a virtual environment due to privacy and sometimes even the associated stigma and prejudice.^55^ In the case of studies on the prevalence of PE among MSM, most studies in the literature were conducted partially or completely through the internet. We chose Facebook and WhatsApp based on the evidence that the use of social media channels for recruiting participants in health research allows lower costs, permits shorter recruitment periods, and improves participant selection in young and hard-to-reach populations, in comparison with traditional methods.^56^

There is evidence that the application of questionnaires over the internet substantially reduces social adequacy bias (in which the participant takes into account social norms when answering the questions) and increases the answers to more sensitive questions.^54^ Studies suggest that in situations of anonymity (as in prevalence studies conducted over the internet), the likelihood of a true answer to sensitive questions is much greater than in situations where confidentiality is guaranteed (participants’ individual data will not be disclosed, but the researcher knows who answered which questionnaire).^57^ As a result of the lower risk of social adequacy bias and the greater probability of providing truthful answers, questionnaires applied over the internet are very appropriate for studies on sensitive topics such as sexual behavior, weight, and use of illicit substances, for example.^58^^,^^59^

The cross-sectional design of our study does not allow setting a cause-and-effect relationship between PE and associated variables. Another limitation is that we did not include questions to assess comorbidities such as erectile dysfunction, prostatitis, depression, and other chronic diseases, nor did we inquire about HIV status or antiretroviral medication use. We also did not ask about condom use. We decided not to use such questions so that the questionnaire would not take too long to respond, since our focus was on the characteristics of sexual behavior. We additionally did not use very strict inclusion criteria and imposed no restrictions related to stable relationships, to obtain a sample as close as possible to the MSM population who could face PE and seek health care for this condition.

We conducted an e-survey without controlling self-selection bias, and we could not calculate our response rate. We know that access to the internet in the metropolitan region of São Paulo was almost 80% at the time of data collection, according to the last Brazilian census (carried out in 2010; https://www.ibge.gov.br) and the Surveillance System for Protective and Risk Factors via Telephone Survey (Vigitel; http://www2.datasus.gov.br). However, the sample studied is younger, more educated, and more sedentary and has a higher income and a higher proportion of White people than the male population aged ≥18 years. Thus, it is not possible to generalize the prevalence estimates of PE obtained in this study to the MSM population living in the metropolitan region of São Paulo.

## Conclusion

PE is a frequent problem among MSM. The prevalence of PE among MSM varies according to the instrument used for the assessment, and the agreement among the 3 assessments was only fair. Combining different tools to investigate PE allows the identification of different characteristics associated with this condition and may result in an improvement in the care of MSM patients.
